# Do Males Form Social Associations Based on Sexual Attractiveness in a Fission-Fusion Fish Society?

**DOI:** 10.1371/journal.pone.0151243

**Published:** 2016-03-17

**Authors:** Anne-Christine Auge, Heather L. Auld, Thomas N. Sherratt, Jean-Guy J. Godin

**Affiliations:** 1 Department of Biology, Carleton University, Ottawa, Ontario, Canada; 2 Department of Cellular and Molecular Medicine, University of Ottawa, Ottawa, Ontario, Canada; The University of Texas at Austin, UNITED STATES

## Abstract

Recent theory predicts that males should choose social environments that maximize their relative attractiveness to females by preferentially associating with less attractive rivals, so as to enhance their mating success. Using the Trinidadian guppy (*Poecilia reticulata*), a highly social species, we tested for non-random social associations among males in mixed-sex groups based on two phenotypic traits (body length and coloration) that predict relative sexual attractiveness to females and sexual (sperm) competitiveness. Based on a well-replicated laboratory dichotomous-choice test of social group preference, we could not reject the null hypothesis that focal males chose randomly between a mixed-sex group that comprised a female and a rival male that was less sexually attractive than themselves and another mixed-sex group containing a sexually more attractive male. The same conclusion was reached when females were absent from the two groups. As might be expected from these laboratory findings, free-ranging males in the field were not assorted by either body length or colour in mixed-sex shoals. The apparent lack of an evolved and expressed preference in wild male guppies from our study population to form social associations with other males based on their relative sexual attractiveness and competitiveness might be due to the fission-fusion dynamics of guppy shoals in nature. Such social dynamics likely places constraints on the formation of stable phenotype-based social associations among males. This possibility is supported by a simulation model which assumes group departure rules based on relative body size and coloration in males.

## Introduction

Animal societies are commonly structured into social groups or networks of non-randomly interacting individuals that preferentially associate with certain social partners, and avoid others, based on their particular phenotypic traits [1–3]. In so doing, individuals can effectively modify their immediate social environment or create social niches, which in turn may influence the selection regime they experience and ultimately their fitness [4–8]. Non-random social associations and interactions can generate covariation between phenotypes of interacting individuals, which is necessary for social evolution [5–7]. Knowledge of social partner-choice behaviour is therefore important for understanding adaptive behavioural decisions, the structure of animal societies and socially-mediated evolutionary change in populations [2–4, 6, 7, 9]. Because social traits are both targets and agents of selection in general [5–7], there is thus increasing interest in how the social environment shapes the evolution of social behaviour (e.g. [8, 10, 11]).

Mating behaviour is social in nature. An individual’s ability to locate receptive mates, compete for mating opportunities and its choice of mating partners has significant fitness consequences and implications for the direction and strength of sexual selection [12]. These mating-related processes can be influenced by the social environment, including local density, sex ratio and the distribution of phenotypes for example (e.g. [12, 13]). In mating systems wherein males and females occur together in groups, females have opportunities to compare males prior to choosing a mate [12]. In such systems, males should in theory [14] actively choose a social environment in which they appear to be relatively more sexually attractive to prospecting females, and potentially more sexually competitive, than their nearby rivals, thereby enhancing their chances of mating and modifying sexual selection. Four recent studies lend support to this hypothesis. Oh and Badyaev [15] observed that less colour-ornamented male house finches (*Carpodacus mexicanus*) in a wild population changed social associations with distinct social groups more frequently than their more ornamented rivals and, in doing so, increased their sexual attractiveness relative to other males in the same flock and achieved greater pairing success than less socially-labile individuals with similar sexual ornamentation. Free-living male forked fungus beetles (*Bolitotherus cornutus*) are negatively assorted by body size within social groups compared to that expected by chance, such that large males with small male social partners enjoy the highest copulation success [16]. Callander et al. [17] experimentally showed in the field that individual territorial male fiddler crabs (*Uca mjoebergi*) were more likely to attract females if they courted immediately alongside smaller, and sexually less attractive and competitively inferior, neighbouring males. In a laboratory experiment, Gasparini et al. [18] found that male Trinidadian guppies (*Poecilia reticulata*) preferentially associated with females that were surrounded by relatively drabber competitors, and that the magnitude of a male’s preference was inversely related to his level of body colour ornamentation. Although not directly related to the above hypothesis, an earlier laboratory study by Dugatkin and Sargent [19] reported that male guppies tended to avoid other males that they had previously seen in (staged) proximity of a female and preferentially associated with males previously seen to be more distant from females. With the exception of these few studies, the hypothesis of social environment choice by males based on their relative sexual attractiveness has not been widely tested empirically, and particularly so in the wild, to our knowledge. There is thus scope for additional studies to confirm its generality.

Here, using the Trinidadian guppy as a model system, we tested the hypothesis of non-random social association among males based on not one but two phenotypic traits (body length and coloration) that indicate their relative sexual attractiveness to females and functional fertility (= success of ejaculates in fertilizing eggs; *sensu* [20]). We predicted that male guppies should prefer to associate with sexually less attractive, and potentially less sexually competitive, rivals when searching for females, and that such social partner choice should be reflected in non-random, phenotype-dependent assortment among males within groups in the wild.

The guppy is a suitable model species for the current study for the following reasons. Guppies are very social animals and in the wild live in fission-fusion societies comprised of highly dynamic, mixed-sex shoals of individuals that are organized into social networks [21, 22]. Males are more mobile than females, moving frequently between shoals in search of mates [21, 23]. Fission-fusion populations, in which groups break up (fission) and reassemble (fusion) frequently, are highly suitable systems for examining ecological correlates of sociality because they respond rapidly to changing conditions [24, 25]. This species has a promiscuous, non-resource-based mating system wherein males experience strong sexual and sperm competition in nature [26–28]. Males are not territorial and show little overt aggression towards each other in the wild ([26], personal observations). Mating typically occurs within shoals of varying membership size and sex ratio [26, 29]. Adult males are ornamented (multiple colours on the body) and smaller than adult females, which are not ornamented and uniformly pale olive [26]. Both males and females exhibit mate choice, with males preferring larger and sexually receptive females [30–32] and females generally preferring to mate with the more brightly coloured and(or) larger of available males [26, 33, 34]. Male mating behaviour is sensitive to the social environment, as characterized by local density, sex ratio and presence of competitors for example (e.g. [18, 35–41]). The colour ornamentation and body length of males correlate positively with their functional fertility [42–47]. We therefore used the body length and colour of males as surrogates for their relative sexual attractiveness and potential sexual (sperm) competitiveness in the current study.

To test our hypothesis and associated prediction, we first quantified the body length and colour ornamentation of individual free-ranging males in a natural population in Trinidad to characterize their respective frequency distribution, because variance in phenotypic traits is a necessary condition for selection to occur and is known to affect male mate-searching and sexual behaviour [48, 49] and the strength of female mating preferences [50] in the guppy. Second, we quantified under standardized laboratory conditions the social preferences of individual males for either of two mixed-sex groups that each contained a rival male that was either more or less colourful and larger or smaller than itself. Third, we collected several free-ranging mixed-sex shoals of guppies in our study population in Trinidad and ascertained whether males occurring in these shoals were associated with one another based on either their body length and(or) body coloration more so than expected by chance using a Monte Carlo randomization model. Lastly, we developed a simulation model to investigate whether phenotype-based, non-random associations among males could emerge within mixed-sex groups over time under certain conditions, particularly under differential movement of males between groups based on their phenotypes, and then carried out a sensitivity analysis of the model to confirm the robustness of its results.

## Materials and Methods

### Ethics Statement

All work reported herein was conducted in accordance with the laws of Canada and of Trinidad and Tobago and the guidelines for use of animals in research of the Canadian Council on Animal Care, and was approved by the Carleton University Animal Care Committee (protocol 13281). Fish were lightly anesthetized in a solution of MS-222 for photography (under approved standard operating procedure protocol VM003). Wild fish used in our study were collected under a permit from the Director of Fisheries, Fisheries Division, Government of Trinidad and Tobago.

### Subjects

Our study subjects were adult guppies collected from the Upper Aripo River (Naranjo tributary), Trinidad, West Indies (10°41’70”N, 61°14’40”W), a low-predation population [29], between April and June 2013. This small uplands river does not contain any major fish predators of adult guppies.

### Measurement of Fish Phenotypic Traits

We measured the body length and quantified the body coloration pattern of all males, and the body length of stimulus females, used in our study in the same manner as follows. Each individual fish was lightly anaesthetized (with MS-222 at 1:10,000 dilution), placed on a piece of white Plexiglas, and its left side photographed along with a metric scale using a 10-megapixel digital camera (Olympus Stylus Tough 6000). Following photography, the fish were placed in a container of aerated water to recuperate. The total body length (to the nearest 0.1 mm) and body colour score of each male, and the total body length of each stimulus female, were later quantified from their photographs using *ImageJ* (http://rsb.info.nih.gov/ij/). Sexual ornamentation in male guppies consists of black (melanin) and red-orange-yellow (hereafter orange) pigment patches and iridescent colours on their bodies [26]. We focused here on measuring orange and black coloration in males because these are the colours most strongly sexually selected in natural guppy populations [26, 34]. We took appropriate measures to standardize our fish photography as much as possible and thus to minimize any variation in male body colour scores possibly introduced by the conditions under which individual males were photographed. For male colour measurements in the laboratory, the ambient lighting conditions were relatively consistent (there was some diffuse natural light entering the lab from small windows located high on one wall), the camera setting was fixed, and the positioning of the camera above the subject was standardized across individuals. For male colour measurements in the field, ambient lighting conditions varied naturally by site and time of day and the camera aperture was adjusted accordingly to standardize image brightness as much as possible, and the positioning of the camera above each subject was standardized as in the laboratory. To avoid inter-observer error, only one of us (A.-C.A.) measured the body coloration and length of males and the body length of females.

We quantified the total area of each male’s body (excluding the fins) and the areas of black and orange spots on his left side. A male’s coloration pattern was then expressed as the proportion of his body’s lateral area (excluding fins) covered by black and orange colour spots, which is hereafter referred to as a male’s ‘colour score’. The body length and colour scores of each male were measured twice (on separate days). As concordance of repeated measures was very high (Pearson’s correlation, *r* = 0.955 for body length, *r* = 0.918 for body colour), they were averaged for each male for use in further analyses. The relative areas of orange and black coloration on the body of individual Upper Aripo males are positively correlated (*r* = 0.126, *N* = 575, *p* < 0.01). Males with longer body lengths and higher body colour scores were deemed more sexually attractive and sexually competitive than smaller and duller males, because female Trinidadian guppies tend to prefer larger and more colourful males as mates in our study population ([51], H.L. Auld, R. Pusiak and J.-G.J. Godin, submitted manuscript), as well as in other Trinidadian populations [26, 34], and male functional fertility is positively correlated with colour ornamentation and body length [42–47].

### Laboratory Experimental Protocols

#### Experiment 1: Social environment preferences

The guppies used in this laboratory experiment were collected haphazardly by hand seining in the Upper Aripo River (Naranjo tributary) and transported in buckets to a laboratory at the University of the West Indies (St. Augustine campus), Trinidad where the experiment was carried out. They were held in mixed-sex glass aquaria filled with aged tap water (24–26°C) and exposed to overhead fluorescent lighting and diffused natural sunlight on a 12 h L: 12 h D cycle, and fed commercial flake food and live brine shrimp (*Artemia franciscana*) twice daily. The fish were acclimatized to these housing conditions for at least one day prior to being used in behavioural trials.

**Fig 1:**
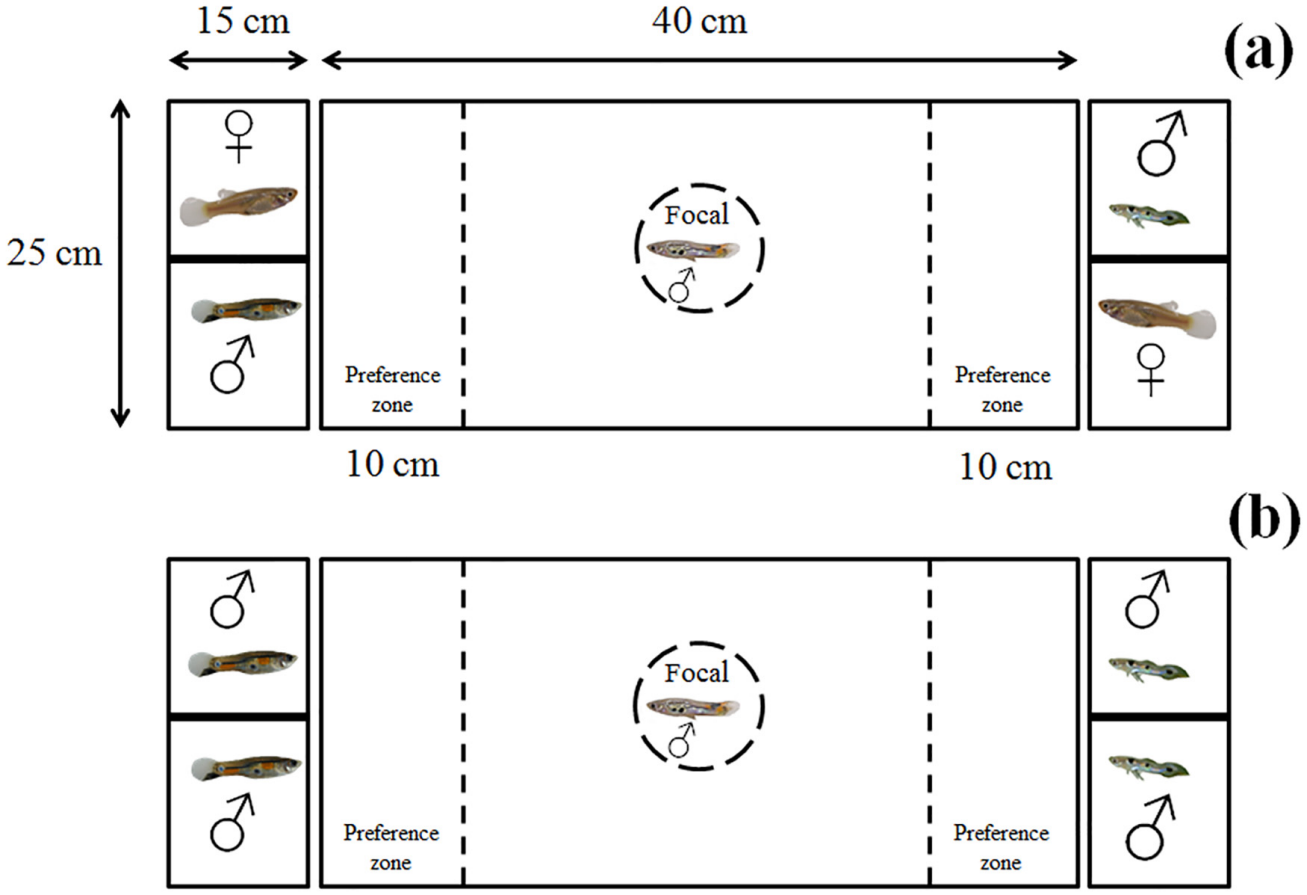
Experimental apparatus. Top-view schematic representation of the experimental apparatus (not to scale), consisting of a central test aquarium holding the focal male and two end compartments holding either (a) a stimulus male and female each in Experiment 1 or (b) two stimulus males each for Experiment 2. During the acclimation and viewing periods (totaling 20 min), the focal male was held in a clear Plexiglas cylinder (represented by the stippled circle) in the centre of the test aquarium. The back line dividing each end compartment denotes an opaque Plexiglas partition preventing the stimulus male and female from interacting socially with each other. The stippled lines denote 10-cm wide preference zones at each end of the test aquarium.

Using a dichotomous-choice apparatus (Fig 1a), we tested for socio-sexual environment preferences in wild-caught males by presenting individual males with a simultaneous choice between two mixed-sex stimulus ‘shoals’, each consisting of one female and one male sexual rival who was either more or less sexually attractive than the focal male. In nature, male Trinidadian guppies sexually pursue females within small mixed-sex groups or shoals comprising most commonly of two or more females and two or more rival males [39]. Therefore, we attempted to simulate such a socio-sexual context here by presenting individual focal males with a simultaneous dichotomous choice between two mixed-sex shoals of conspecifics that differed in the phenotypes of rival males within them. We predicted that a focal male on average would prefer to socially associate with the shoal containing the rival male that was less colourful and(or) smaller than itself.

The experimental apparatus consisted of a central Plexiglas aquarium (40 x 20 x 25 cm; L x W x H), flanked by a clear Plexiglas compartment (15 x 20 x 25 cm) on each end (Fig 1a). The area within 10 cm from each end of the test aquarium was delineated as a preference zone. This distance is equivalent to four male guppy body lengths, which falls within the range of inter-individual distances observed in wild fish shoals [52]. The central test aquarium held a focal male, and each of the two end compartments held one stimulus rival male and one stimulus female, separated by an opaque Plexiglas partition to prevent behavioural interactions between them. The two stimulus males were placed diagonally across from each other in the two end compartments. This arrangement of holding each fish in separate compartments allowed for testing of the social association preference of the focal male based on visual cues only. The bottom of the central test aquarium and end compartments was covered with natural river gravel. The back and front walls of the aquarium and three sides of each of the end compartments were covered with brown paper to reduce any external disturbances for the test fish. The apparatus was illuminated overhead as for the holding aquaria.

In any given behavioural trial, the two stimulus females were matched for body length (mean ± SE difference = 0.86 ± 0.05 mm) and were gravid (pregnant) and thus sexually unreceptive [26]. Although male guppies prefer to mate with sexually receptive females over gravid and unreceptive ones when given the choice [30, 31], they will nonetheless sexually pursue, court and attempt to mate with previously-mated gravid females in both the wild and in the laboratory [26, 30–32, 53] and can successfully inseminate unreceptive gravid females through forced sneak copulations [30, 54, 55] and sire offspring [27, 28]. We used gravid females as stimulus fish here to minimize any influence of female sexual activity on male social association behaviour and because most adult females in natural guppy populations in Trinidad are inseminated and pregnant at any given time [26–28]. Because male guppies prefer to mate with larger females [32, 39, 53], this latter procedure standardized the reproductive state of the paired stimulus females and thus ensured that male social associations would not be confounded by any differences in female size and sexual behaviour towards focal males. The two stimulus males were chosen to differ in their total body length and(or) amount of body coloration, and thus their relative sexual attractiveness to females and potential sexual competitiveness. When choosing the three males for each behavioural trial and prior to placing them into the apparatus, we initially measured them (without anesthesia) using a metric measuring board and categorized their overall body coloration by eye as either similar, more colourful or less colourful relative to each other. At the end of the trial, each male was photographed digitally for accurate measurements of body length and coloration, as described below. Since guppies can become familiar with each other after 12 days of association [56] and familiarity can potentially affect social associations [57] and mate choice [58], the focal male, stimulus males and stimulus females used in any given trial were taken from different holding aquaria and were presumably unfamiliar with each other prior to testing. We cannot exclude the possibility of social familiarity among fish used in any given behavioural trial owing to their (unknown) natural social experiences prior to being collected in the field. However, this is unlikely because our test fish were collected from several pools along the Upper Aripo River (Naranjo tributary) over a period of about two months, male and female guppies move between pools and shoals in the wild [21, 23], and the collections were mixed and the fish randomly placed in several holding aquaria in the laboratory upon arrival from the field. Even if this possibility of prior social familiarity occurred, it would simply add noise rather than confound our results.

In choosing wild-caught focal and stimulus males for the behavioural trials, we aimed to represent the natural range of body lengths and coloration of free-ranging males in the Upper Aripo River (see Results) and to present each focal male with two rival stimulus males (one in each of two stimulus shoals) that were different to varying degrees in body length and coloration relative to each other and to the focal male in one of three treatments as follows. The paired stimulus males either differed in both body length and coloration from each other; that is, one male was both more colourful and larger than the other (Treatment 1, *N* = 35, Table 1), were matched for body length but differed in overall body coloration (Treatment 2, *N* = 42), or were matched for overall body coloration but differed in body length (Treatment 3, *N* = 24). In all three treatments, the focal males were intermediate in length and colour between the paired stimulus males that differed in body length and(or) colour from one another, or they were similar to the paired stimulus males in either body length or colour in trials when the latter males did not differ from each other in either length or colour (Table 1). The behavioural trials were run blindly of the treatments. At the end of the experiment, we confirmed the treatment category of each behavioural trial by measuring the differences in body length and colour scores of the paired stimulus males in the trial using their individual digital photographs (as described below). Paired stimulus males were deemed similar in body length if the difference in their total body length was ≤ 1 mm and similar in coloration if the difference in their colour score (proportion) was ≤ 0.01; otherwise, they were deemed different in these phenotypic traits. Therefore, for each treatment, focal males were individually given a choice to socially associate with either a ‘less attractive’ stimulus shoal (containing a rival male that was either smaller and(or) less colourful than the focal male) or a ‘more attractive’ stimulus shoal (containing a rival male that was larger and(or) more colourful than the focal male). The average (± SE) body length and colour score of all males used in this experiment was 25.2 ± 0.1 mm (range = 19.8–31.9) and 0.061 ± 0.001 (0.001–0.150), respectively, and thus near the median for both traits in wild males haphazardly collected in the Upper Aripo River (see Results).

**Table 1:** Body length and body colour scores of males used in Experiment 1. Mean ± SE total body length (mm) and body colour score (proportion of left side of body covered with black and orange colours) of focal male guppies and the paired stimulus males categorized as relatively more attractive (MA) and less attractive (LA) used in each treatment of laboratory Experiment 1. The body length and colour scores of paired stimulus males in each treatment were separately compared with the paired *t*-test. *N* denotes the number of replicated trials in each treatment.

	Trait	Focal male	MA male	LA male	Difference |MA—LA|	Paired *t*-test
Treatment 1 (*N* = 35)	Length	25.3 ± 0.30	26.5 ± 0.31	23.9 ± 0.30	2.57 ± 0.28	*t* = 9.28; *p* < 0.001
	Colour	0.063 ± 0.004	0.081 ± 0.004	0.043 ± 0.004	0.038 ± 0.004	*t* = 9.05; *p* < 0.001
Treatment 2 (*N* = 42)	Length	25.1 ± 0.22	25.4 ± 0.23	25.3 ± 0.22	0.10 ± 0.08	*t* = 1.31; *p* = 0.198
	Colour	0.062 ± 0.004	0.084 ± 0.003	0.028 ± 0.003	0.056 ± 0.004	*t* = 14.42; *p* < 0.001
Treatment 3 (*N* = 24)	Length	24.6 ± 0.42	27.3 ± 0.40	23.3 ± 0.39	4.09 ± 0.30	*t* = 13.60; *p* < 0.001
	Colour	0.059 ± 0.005	0.059 ± 0.004	0.061 ± 0.005	0.002 ± 0.001	*t* = 1.61; *p* = 0.122

All trials followed the same procedure. At the outset of a trial, opaque screens were placed between the end compartments and the test aquarium (Fig 1a). The focal male was then placed in a clear Plexiglas cylinder (7 cm diameter) in the centre of the test aquarium and the stimulus males and females placed in their respective halves of the end compartments. The particular end compartment assigned to hold each stimulus shoal was determined at random. Whilst out of view of one another, the fish were allowed to acclimatize to the aquarium environment for 10 min. After this period and with the focal male still in the central cylinder, the opaque screens were removed and the focal male was allowed to view both mixed-sex stimulus shoals in their respective end compartments for another 10 min. At the end of this viewing period, the cylinder was gently raised and the focal male was allowed to freely choose to associate with either of the stimulus shoals for 15 min. To minimize any external disturbances, the focal male was filmed using a video webcam (Logitech HD Pro Webcam C910) located 30 cm above the test aquarium. The video footage was displayed and observed in real time on the screen of a remote laptop computer. The water in the test aquarium and end compartments was replaced with fresh aged water after every completed trial.

For each 15-min choice trial, the time that the focal male spent associating with (i.e. within ≤ 10 cm of) either stimulus shoals was quantified using *JWatcher*^*™*^ [59]. More specifically, the time that the focal male spent within the preference zone and directly in front of the stimulus females and the adjacent stimulus male separately was quantified as a measure of his preference for either mixed-sex stimulus shoal. Such association or proximity time is a commonly used proxy for social attractiveness [60, 61] and mating preferences in poeciliid fishes (e.g. [39, 62]), and is a predictor of social [63] and mating [53] partner choice in the guppy.

Across the three treatments, we similarly tested a total of 101 focal males that ‘sampled’ both stimulus shoals (entered both preference zones and spent time in front of both stimulus females and stimulus males) during the 15-min observation period. Because of the limited availability of males, approximately 15% of focal males were re-used as stimulus males; however, no male was used more than once as a focal male and never in combination with the same males as previous. At the end of the experiment, the fish were returned live to the Upper Aripo River.

#### Experiment 2: Controlling for sexual context

Because we hypothesized that male guppies should actively choose their social environment in a manner that enhances their relative sexual attractiveness to females and sexual competitiveness vis-à-vis rival males, we carried out a corollary control experiment in the laboratory (at Carleton University) to ascertain whether any shoal preference exhibited by focal males in Experiment 1 could be owing to a sexual context (i.e. presence of females) and not simply to focal males exhibiting a non-sexual, social preference for or avoidance of male conspecifics based solely on their phenotypes (i.e. body length or coloration). This experiment was carried out blindly of the results of Experiment 1.

We used adult guppies that were laboratory reared, first- and second-generation descendants of adults collected the Upper Aripo River, because we had too few wild guppies available for this particular experiment. We used the same fish holding conditions, dichotomous-choice apparatus and general protocol described for above experiment, except that we removed the sexual context by replacing the stimulus females in the two end compartments of the apparatus with males (Fig 1b). Therefore, individual focal males were presented with a choice to socially associate with either of two (same-sex) shoals of males that were individually placed in either one of the two end compartments of the apparatus determined at random. This is not an unnatural scenario; although most guppy shoals are comprised of males and females in nature, not all are. Solitary male guppies commonly encounter other males as they move between shoals in nature [21], and such males sometimes will temporarily form small same-sex groups before dispersing in search of females ([21], S.J. Potter, H.L. Auld, T.N. Sherratt and J.-G.J. Godin, unpublished data).

One of the paired stimulus shoals consisted of two males that were similar in body length and coloration to each other, but both larger and more colourful than the focal male; this shoal is hereafter referred to as the more attractive shoal. In comparison, the other stimulus shoal (i.e. the less attractive shoal) consisted of two males that were similar in body length and coloration to each other, but both smaller and less colourful than the focal male. Subsequent measurements of male body length and colour scores from their digital photographs (following the same procedure as described above) confirmed that the categorized more attractive stimulus males (*N* = 60), less attractive stimulus males (*N* = 60) and focal males (*N* = 30) significantly differed in body length (one-way ANOVA, *F*_2,147_ = 87.56, *p* < 0.001) and colour scores (*F*_2,147_ = 57.79, *p* < 0.001) from each other (S1 Fig).

During each 15-min choice trial, the focal male was observed through a small viewing window in a blind placed in front of the aquarium to minimize external disturbances. Following the same protocol as for Experiment 1, we recorded the time that the focal male spent associating with each same-sex (male) stimulus shoals, as a measure of his preference for either shoal. We tested a total 30 focal males that sampled both stimulus shoals. Focal males were used only once, whereas some stimulus males were re-used once but never in combination with the same males as previous. In the absence of females in their immediate social environment, we predicted that focal males would exhibit no preference for either of the two same-sex (male) stimulus shoals.

### Statistical Analyses of Experimental Data

To meet the assumption of normality of data distribution for both experiments, the proportion of total association time that focal males spent near each of the paired stimulus shoals, and the proportion of time spent near the male and female within each stimulus shoal (in Experiment 1), in each trial were arcsine transformed before analysis. All statistical tests were carried out in the R statistical environment [64].

For Experiment 1, we first tested for each treatment separately the prediction that focal males would spend proportionally more time associating with the less attractive stimulus shoal than that expected by chance using the one-sample *t*-test. Second, we ascertained whether focal males spent proportionally more time near the female than the male in each stimulus shoal than expected by chance using the one-sample *t*-test. The expected proportion value for no choice (i.e. chance) is 0.50. Third, using linear regression analysis, we tested whether the proportion of time focal males spent associating with the less attractive shoal in Treatment 2 covaried with the colour score of the focal male and(or) the difference in the colour scores of the more and less attractive males in the paired stimulus shoals in a trial. Similarly, we tested for covariation between the association time for the less attractive shoal in Treatment 3 and the body length of the focal male and(or) the difference in the body length of the more and less attractive males in the paired stimulus shoals in a trial. Lastly, using a general linear model, we tested for overall effects of the differences in the body length and body colour score of the paired stimulus males, and their interaction, on the magnitude of the focal males’ preference for the less attractive shoal (as measured by proportion of total association time) across all three treatments (*N* = 101).

For Experiment 2, we first tested the null hypothesis that the proportion of time that the focal males spent associating with either of the two same-sex (male) stimulus shoals would not differ significantly from chance using the one-sample *t*-test. Second, using linear regression analysis, we tested whether the proportion of time focal males spent associating with the less attractive stimulus shoal covaried with the body length and colour score of the focal male and(or) the differences in the mean body length and mean colour scores of the more and less attractive males in the paired stimulus shoals in a trial. Because the two males in the less attractive shoal were matched for both their body length and colour, and similarly for the two males in the more attractive shoal, we used the differences in the averages of their body length and colour score respectively as covariates.

### Male-Male Social Associations in the Wild

We collected guppy shoals in the Upper Aripo River (Naranjo tributary) to test whether males occurring in mixed-sex social groups in the wild are assorted non-randomly by body length and(or) body coloration. If individual males actively seek a social environment (shoal) in which they are more sexually attractive and(or) more sexually competitive than their nearby rivals, then we expected that they would show non-random patterns of association based on body length and(or) colour within shoals in the wild. This part of our study was carried out blindly of the results of laboratory Experiments 1 and 2 above.

We observed free-ranging guppy shoals in the Upper Aripo River between 09:00 and 15:00 hrs from vantage points on the shoreline of six adjacent pools along a 100-m stretch of the river on 10 days between 24 April and 17 June 2013. Individual wild guppies are capable of dispersing at least this distance, either upstream or downstream, over shorter periods [21]. The selected pools were shallow (< 1 m depth), ranged in surface area from approximately 1 to 50 m^2^ and were between 1 and 41 m apart from each other, interconnected by shallow riffle areas (S2 Fig) that allowed fish movement between them. Each pool was visually scanned for the presence of mixed-sex guppy shoals that consisted of two or more males sexually pursuing at least one female within the shoal [39]. Each visually-localized focal shoal, defined as fish within three body lengths of each other [52], was captured in its entirety with a hand seine (3 m x 1 m) and placed in a bucket containing river water. Adult males and females in each collected shoal were then enumerated and individually photographed with a digital camera in the field using the same procedure described above. Following photography, the captured fish were retained in buckets with aerated river water and only released back into the river (at the site of their collection) at the end of the day to avoid re-capture and pseudo-replication. To provide sufficient time for fish to move within and between pools and to reconstitute social associations between samples and thus to further minimize the likelihood of pseudo-replication, we collected a maximum of two focal shoals per pool per sample day, one in the morning and one in the afternoon (separated by a minimum of 2 h). Wild guppies move more within than between pools and males are more mobile than females [21, 23]. A total of 67 focal mixed-sex guppy shoals, containing a total of 177 male guppies, were collected. On average (± SE), the shoals comprised 5.53 ± 0.40 fish (range = 3–19), of which 2.63 ± 0.12 (2–6) were males and 2.90 ± 0.35 (1–15) were females.

The total body length and body coloration pattern of individual males that occurred in the collected mixed-sex shoals were later quantified from their photographs using *ImageJ*, as described above. Their body length and colour score averaged 24.8 ± 0.1 mm (18.9–29.8) and 0.062 ± 0.002 (0.001–0.137), respectively. Based on our visual inspections of the photographs of all males collected and the data on their individual lengths and colour scores, we could not detect any males that were identical phenotypically. Therefore, we are confident that we did not repeatedly capture the same male(s) in our shoal collections and that our field-based data are not pseudo-replicated.

The shoal ‘scan’ sampling method we used here assumes that all individuals present in an intact group at the time of sampling were socially associated with each other for some (unknown) time prior to their being sampled—the so-called ‘gambit-of-the group’ paradigm [3]. This method is commonly employed and has previously revealed non-random social associations among free-living individuals in the guppy [22, 65, 66] and in other taxa [2, 3].

To determine whether males occurring in mixed-sex wild shoals were more or less assorted either by body length or coloration than expected by chance, we first calculated the inter-male variation in both body length and colour scores within shoals using the within-group variance component of the *F*-statistic obtained from an analysis of variance (ANOVA). We then ascertained whether the within-group variance values obtained from the data on male length and coloration separately differed from the respective within-group variance values expected by chance using Monte Carlo-type randomization of group membership [3, 67, 68]. We simulated 10,000 random guppy shoals using MATLAB [69]. In the simulations, the number of wild guppy shoals collected (*N* = 67) and the observed membership size of each shoal were conserved, and all individual males (and thus their respective body length and body coloration scores) in the collected wild shoals were randomly shuffled and assigned to newly-formed simulated shoals. That is, male-male associations within shoals were randomized and reshuffled across all 67 shoals during each simulation run. For each of the 10,000 simulated shoals, the within-group variance of male body length and coloration was calculated separately using an ANOVA as described above. The observed within-group variances for the body length and coloration of males present in the collected wild shoals were then compared to the frequency distribution of the 10,000 simulated variance values for each of these two phenotypic traits separately using R. For a two-tailed test, an observed value of variance must fall in either the lower or upper 2.5% of the distribution of simulated values for the rejection of the null hypothesis that the observed associations of wild male guppies in mixed-sex shoals, based on either their body length or coloration, was generated by chance [3, 67].

### Modeling Male-Male Associations in Mixed-Sex Groups

To ascertain whether preferential social associations by males can generate non-random patterns in body length and coloration characteristics within social groups, we constructed a simulation model in R of a fission-fusion population comprising of a pre-determined number of mixed-sex groups. In the model, we assumed a multi-group system in which the probability of any male leaving his current group was inversely related to his phenotype (body length and colour score) relative to the phenotype of other male members of the group and the male/female sex ratio of the group, and we simply evaluated the long-term consequences of these movement rules. This assumption is based on the theory [14] and empirical findings [15] that less ornamented males would benefit more than highly ornamented rivals by moving more in search of social environments in which their relative sexual attractiveness and(or) competitiveness, and thus mating success, are maximized.

**Fig 2:**
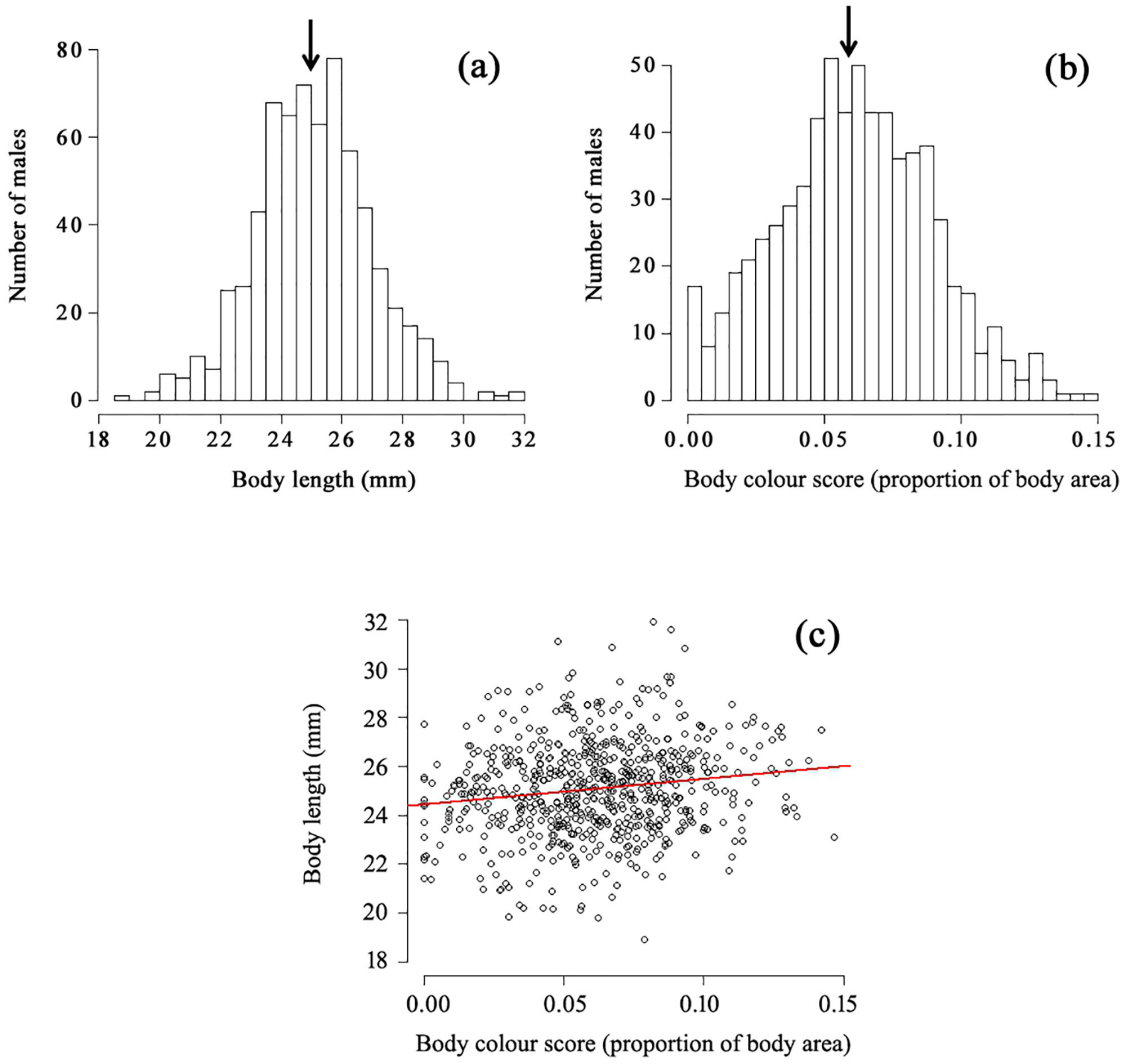
Phenotypic traits of wild-caught males. Frequency distribution of (a) total body length (mm) and (b) body colour scores (proportion of body area covered with orange and black colours) of wild male guppies (*N* = 672) collected haphazardly by hand seining in the Upper Aripo River, and the correlation (*r* = 0.152, *p* < 0.001) between the body length and colour score of individual males (c). Arrows in panels (a) and (b) indicate the mean body length and body colour score of the focal males used in Experiment 1.

The model starts with a fixed number of groups (here *N* = 67, reflecting our field data) and a fixed number of females per group (set at 2 throughout), which are assumed to stay in their groups. A fixed number of males (*N* = 170, reflecting our field data) were then randomly allocated over the available groups. The body coloration scores and body length of these males were drawn from a multivariate normal distribution (default parameters: mean colour score = 6, standard deviation of colour = 3; mean length = 24, standard deviation of length = 1.5; correlation between colour and length, *r* = 0.15; reflecting our field data (see Fig 2c)). For each time step, *N* random selections of males are made (so that each male is chosen on average once per time step) for a potential movement. When a randomly chosen individual *i*, belonging to group *g* with mean coloration ${\overset{-}{c}}_{g}$, mean size ${\overset{-}{s}}_{g}$ and sex ratio $R_{g}$ is considered, then it is moved to another randomly chosen group with probability *p*_*move*_ which is set according to a simple sigmoidal (logistic) function:

$$p_{move}=\frac{\text{exp}\left(a\right)}{1+\text{exp}\left(a\right)}$$

where

$$a=\left\{\alpha +\;\;\beta_{c}\left({\bar{c}}_{g}-c_{i,g}\right)+\beta_{s}\left({\bar{s}}_{g}-s_{i,g}\right)+\beta_{R}R_{g}\right\}$$

Here, ∝ is the intercept and *ß*_C,_ *ß*_*S*_ and *ß*_*R*_ are the rate parameters assumed positive for colour, length and sex ratio-based movement, respectively. As such, the probability of movement is higher for individual males that are relatively dull in coloration compared to the mean of their particular group, and higher for those individuals that are smaller on average compared to others in the group. The movement rate of males is also greater for those males that find themselves in groups with higher male/female ratios. Each and every time a potential movement is considered, the phenotypic attributes of movers and non-movers are recorded and (if a movement has taken place) the group compositions are recalculated. At the end of a given time step, when males have had the opportunity to move on average once, the variances in male body colour and body length within groups are calculated just as one would derive in a standard ANOVA. The simulation is then repeated over multiple time steps (here 100) to evaluate whether the within-group estimates of variance either decrease (which would indicate positive assortment of males based on similar phenotypes) or increase (indicating negative assortment based on dissimilar phenotypes) over time as a consequence of accumulated between-group movement of males.

### Sensitivity Analysis of the Model

To test the robustness of the results of our simulation model, we carried out a sensitivity analysis as follows. In general, the parameter values of the model were the same as those used in the original model (see above), except that we systematically and independently varied one of the model’s main parameters at a time, whilst keeping all others constant, to test the model’s sensitivity to the varied parameters. We chose to vary the number of males (*N*) in the ‘population’ and the phenotype-based movement parameters *ß*_*C*_ and *ß*_*S*_. For each value of a varied parameter, the model generated an output of within-group variance in both male body coloration and body length over time. We then regressed these variance estimates against time, which yielded a regression coefficient. A coefficient of 0 denotes no estimated change in the magnitude of within-group variance over time, whereas a positive coefficient represents estimated increasing variance values over time (i.e. increasing negative assortment) and a negative coefficient represents decreasing variance values over time (i.e. increasing positive assortment). The model simulation was repeated five times for each value of a varied parameter value and the regression coefficients produced were averaged for that parameter value. If our model’s results are robust, then we would expect calculated regression coefficients to fluctuate non-systematically about a population mean of 0, across the tested ranges of parameter values.

## Results

### Distributions of Male Phenotypic Traits

Both total body length (Fig 2a) and coloration (Fig 2b) varied continuously among wild male guppies in the Upper Aripo River, approximating normal distributions. There is thus considerable variation in these two male phenotypic traits in this population. Although the body length and colour score of individual males were positively correlated (Fig 2c; *r* = 0.152, *r*^2^ = 0.023, *N* = 672, *p* < 0.001), the correlation is weak and a considerable amount (> 97%) of the observed variation in these two traits is thus not explained by a linear regression model; body length is therefore not a strong predictor of body coloration (as measured here), and *vice versa*, in our study population.

### Experiment 1: Social Environment Preferences

**Fig 3:**
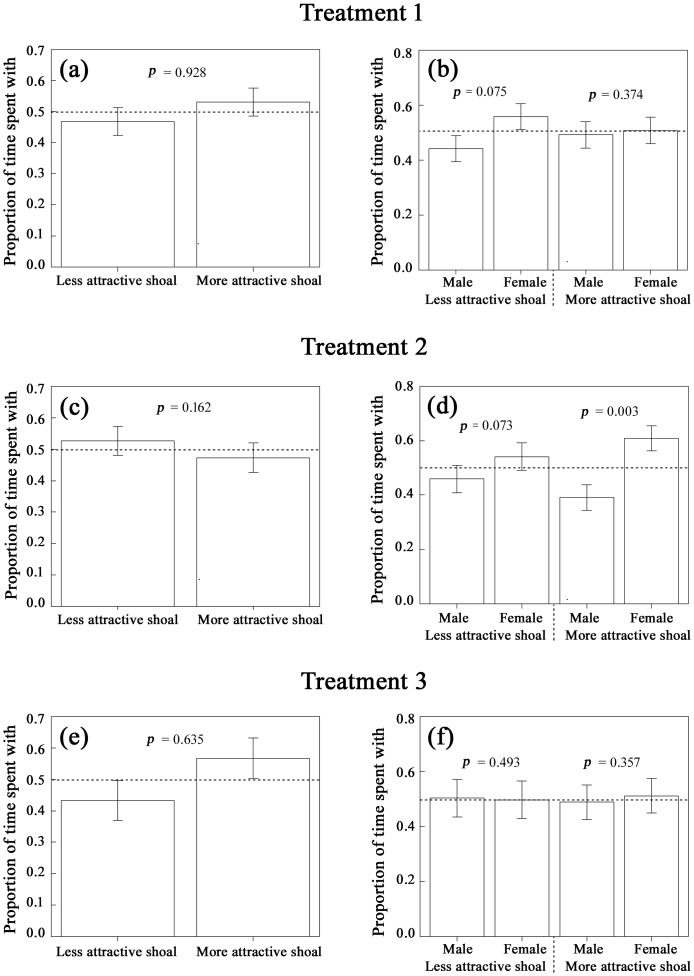
Group association preferences of males in the laboratory. Mean (± SE) proportion of time that focal males spent associating with the paired less attractive and more attractive stimulus shoals, and with the male and female within each mixed-sex stimulus shoal, in Treatment 1 (panels a, b), Treatment 2 (panels c, d) and Treatment 3 (panels e, f) of Experiment 1. The horizontal stippled lines denote random choice (or chance). The *p*-values shown were obtained from one-sample *t*-tests, comparing the observed proportion of association time spent by focal males (*i*) near the less attractive of the two mixed-sex stimulus shoals (panels a, c, e) and (*ii*) near the female in each of paired stimulus shoals (panels b, d, f) against that expected by chance. The male in the less attractive stimulus shoal was either both less colourful and smaller than the male in the more attractive stimulus shoal in Treatment 1, similar in length but less colourful than the male in the more attractive shoal in Treatment 2, or similar in coloration but smaller than the male in the more attractive shoal in Treatment 3 (see Table 1 for details).

**Fig 4:**
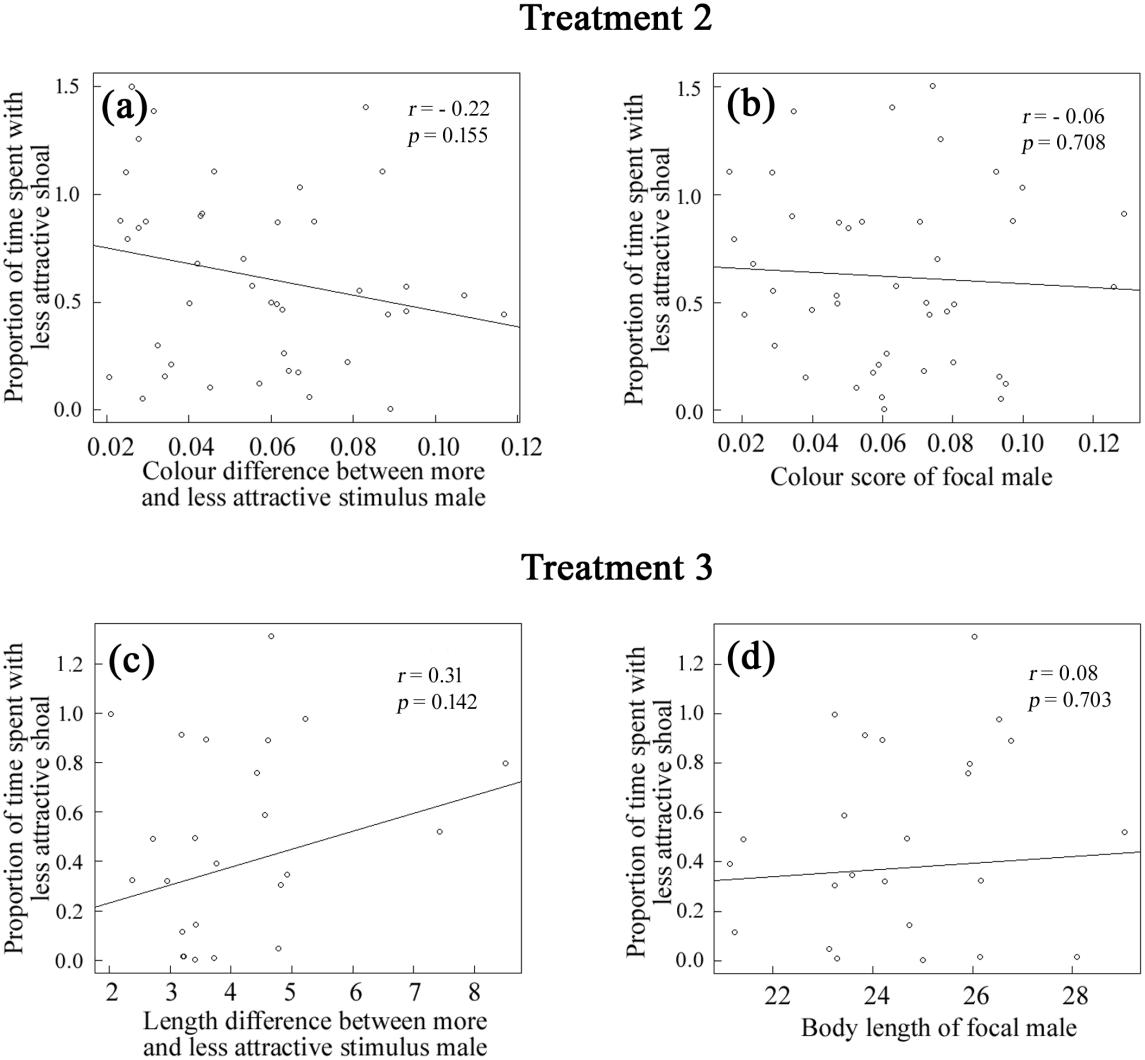
Relationship between social association with the less attractive shoal and the phenotypic differences between stimulus males. Relationships between the observed proportion of association time spent by focal males near the less attractive of the two mixed-sex stimulus shoals and (*i*) the differences in the colour scores (a) and body length (c) of the more attractive and less attractive males in these shoals, and (*ii*) the colour score (b) and body length (d) of individual focal males for Treatments 2 and 3 separately.

Contrary to the hypothesis under test that males should prefer to join social groups in which they are relatively more sexually attractive/sexually competitive than their rivals, male guppies from the Upper Aripo River did not preferentially associate with a less attractive mixed-sex shoal, which contained a less colourful and(or) smaller male rival, over a nearby more attractive shoal, which contained a more colourful and(or) larger male rival (Fig 3a, 3c and 3e). Reflecting such random choice, the proportion of time that focal males spent associating with the less attractive shoal did not covary significantly with the magnitude of the differences in body coloration (Treatment 2, Fig 4a) or body length (Treatment 3, Fig 4c) between the more and less attractive stimulus males in the paired shoals, nor with the body colour (Treatment 2, Fig 4b) or body length (Treatment 3, Fig 4d) of the focal male. Reinforcing these results, the proportion of time that focal males spent associating with the less attractive shoal (Fig 3a, 3c and 3e) was not influenced by the differences in the body length (linear model, *F*_*1*,*97*_ = 0.477, *p* = 0.492) and body colour score (*F*_*1*,*97*_ = 0.037, *p* = 0.849) of the paired stimulus males in individual trials, or their interaction (*F*_*1*,*97*_ = 0.032, *p* = 0.859), across the three treatments.

When randomly associating with either of the paired stimulus shoals (Fig 3a, 3c and 3e), focal males did not spend more time near the female than the male within the shoals in all three treatments (Fig 3b, 3d and 3f), with only one exception (Treatment 2, more attractive shoal; Fig 3d). Males were thus equally attentive to both female and adjacent rival male within the stimulus shoals, suggesting that they were motivated to mate but also concurrently sensitive to potential sexual rivals nearby.

### Experiment 2: Controlling for Sexual Context

The above finding of a lack of expressed social environment preference in Upper Aripo male guppies (Fig 3) is not context dependent. When females in the stimulus shoals were replaced with males in control Experiment 2 (i.e. sexual context removed), the proportion of time that the focal males spent associating with either of the two the same-sex (male) stimulus shoals did not differ significantly from chance (*t*_29_ = 0.423, *p* = 0.675; less attractive shoal, 349.8 ± 38.4 s or 0.454 ± 0.047 proportion time; more attractive shoal, 415.9 ± 37.3 s or 0.546 ± 0.947 proportion time). Reflecting such random choice, the proportion of time that focal males spent associating with the less attractive shoal did not covary with the body colour (*F*_*1*,*24*_ = 1.958, *p* = 0.175) or body length (*F*_*1*,*24*_ = 0.001, *p* = 0.975) of the focal male, nor with the magnitude of the difference in body coloration between the more and less attractive stimulus males in the paired shoals (*F*_*1*,*24*_ = 1.048, *p* = 0.316) in Experiment 2. However, the proportion of time that the focal males spent near the less attractive stimulus shoal (containing smaller and less colourful males) was marginally and negatively influenced the difference in body length between the stimulus males in the paired shoals (*F*_*1*,*24*_ = 4.275, *p* = 0.049), but not so much as to promote a significant preference for the more attractive shoal (containing larger and more colourful males). There was no significant interaction between the differences in body colour and body length of the paired stimulus males on the response variable (*F*_*1*,*24*_ = 1.963, *p* = 0.174).

### Male-Male Social Associations in the Wild

**Fig 5:**
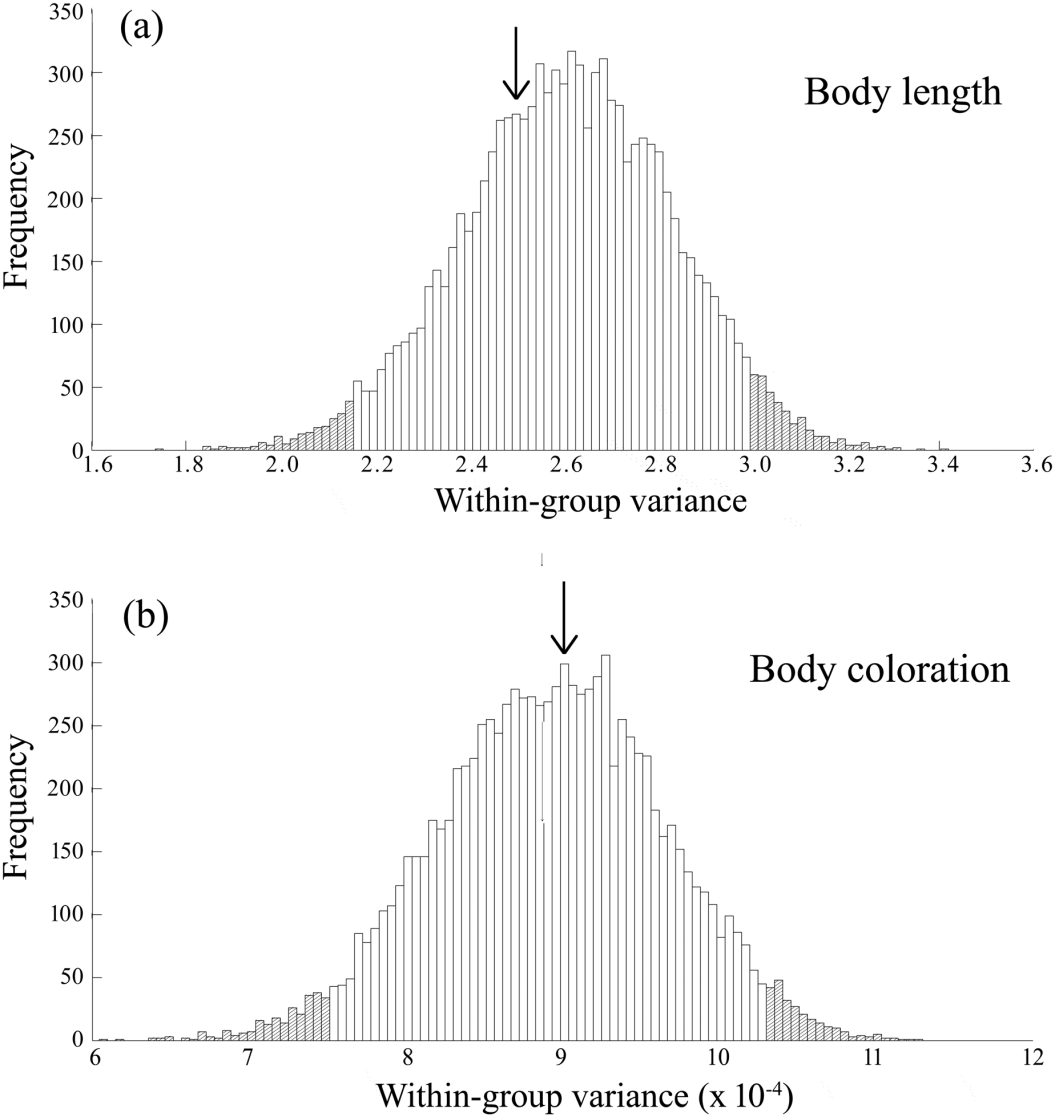
Results of the randomization model. Frequency distribution of within-group variance in body length (a) and body colour score (b) of Upper Aripo River male guppies in 10,000 randomly simulated mixed-sex shoals, as described in more detail in the Materials and Methods. The arrows indicate the actual calculated within-group variance, obtained from an ANOVA, in the body length and body colour score, respectively, of male guppies present in the free-ranging mixed-sex shoals (*N* = 67) that were collected in the Upper Aripo River, Trinidad. The hatched areas of the graphs represent the lower and upper 2.5^th^ percentiles of the frequency distributions of the simulated within-group variance estimates.

Contrary to the theoretical expectation of phenotypic assortment based on differences in sexual attractiveness among males (cf. Introduction), male guppies were not assorted by either total body length or coloration in free-ranging shoals in the wild. Neither of the calculated within-group variance for body length (s^2^ = 2.47) nor body coloration (s^2^ = 8.99 x 10^−4^) fell within the top or bottom 2.5 percentiles of the distribution of the 10,000 randomly generated within-group variance values (Fig 5), suggesting random phenotypic assortment of males within shoals in the wild.

### Modeling Male-Male Associations in Mixed-Sex Groups

**Fig 6:**
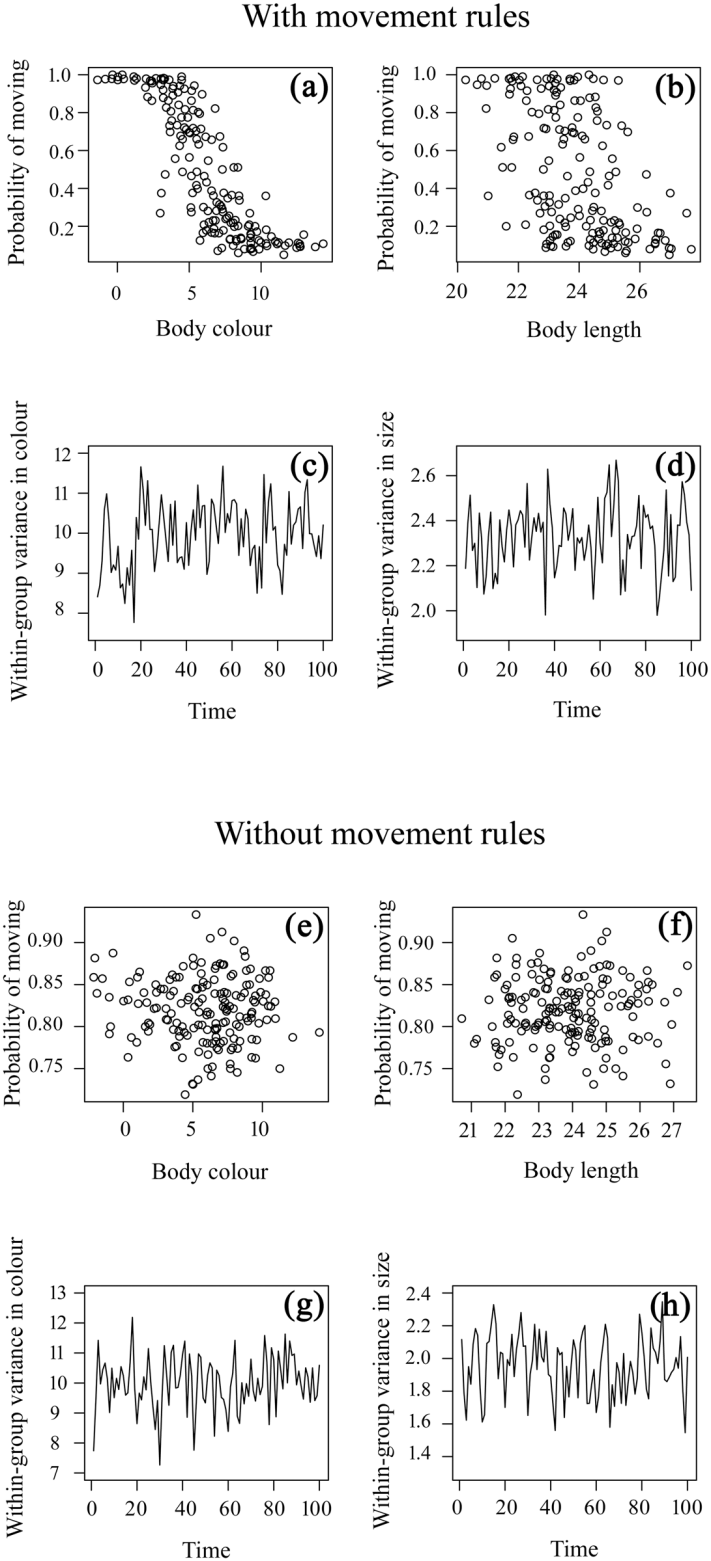
Results of the simulation model. Simulation model output after 100 time steps, with either strong phenotype-dependent male movement [panels (a)-(d), model parameters: ∝ = 0, *ß*_*C*_ = 20, *ß*_*S*_ = 20, *ß*_*R*_ = 1] or no phenotype-dependent male movement [panels (e)-(h), model parameters: ∝ = 0, *ß*_*C*_ = 0, *ß*_*S*_ = 0, *ß*_*R*_ = 1]. Top panels (a-b, e-f) show the realized probability of a male moving out of its current group in search of another group as function of both his body coloration and body length. Bottom panels (c-d, g-h) show temporal fluctuations in the within-groups variance in male body coloration and body length as a result of individual males moving out of their current group with a certain phenotype-dependent probability, as determined by the model’s movement rules depicted in the top panels.

Here, we employed our simulation model simply to confirm that, even with movement of males between shoals being dependent on their phenotypes, stable assortment among males within groups based on either their ornamentation or body size did not emerge under conditions chosen to match our field observations in the Upper Aripo River (Fig 6).

Despite strong phenotype-based movement rules (Fig 6a and 6b), this behaviour had little effect on the within-group variance in phenotypes (Fig 6c and 6d). Furthermore, although the estimates of within-group variance between consecutive time steps are not independent, there is no underlying trend in the magnitude of either estimates of variance over time as a result of accumulated male movement activity. Duller or smaller males are more likely to move (Fig 6a and 6b), but have to go somewhere and typically end up in groups they also have to leave. Clearly with movement rules based on differences from the group average (rather than population average), even colourful or large males may occasionally move if they are in a group with even more colourful or larger males, which may further explain why assortment fails to emerge among males. Likewise, with no phenotype-dependent male movement (Fig 6e and 6f), the within-group variance again estimates the same underlying variability in the population (Fig 6g and 6h), although this time movement is only dependent on sex ratio and not male phenotype. Sensitivity analysis revealed that our model inference is robust, as the above result of a lack of emerging phenotypic assortment among males over time remained unchanged over a range of parameter values (i.e. number of males in the system, and different values of *ß*_*C*_ and *ß*_*S*_) (see S3–S6 Figs).

Collectively, our model simulation results confirm that even strong phenotype-based movement rules at the individual level do not inevitably lead to stable assortment among males within groups based on either their ornamentation or body size at the population level.

## Discussion

Guppies in nature live in mixed-sex, highly promiscuous, fission-fusion social groups [21, 22, 26–28]. Males are more mobile than females and frequently move between shoals in search of sexually receptive females [21, 23]. Within groups, female guppies generally prefer to mate with the more brightly coloured and(or) larger of available males [26, 33, 34], and male mating behaviour is sensitive to the socio-sexual environment, as characterized by local density, sex ratio and presence and phenotype of sexual competitors for example (e.g. [18, 19, 35–41]). If male guppies actively choose to join certain shoals over others to enhance their relative sexual attractiveness to females and their sexual competitiveness and thus mating success, as predicted by theory [14], then we expected that they would prefer to socially associate with relatively less attractive and less competitive sexual rivals than themselves and therefore to be negatively assorted by body coloration and(or) length, two sexually-selected traits in the guppy [26, 33, 34], within free-ranging shoals in nature. However, contrary to our a priori prediction, male guppies from the low-predation Upper Aripo River population in Trinidad did not exhibit an association preference for a mixed-sex shoal containing a less attractive male sexual rival over another nearby mixed-sex shoal containing a more attractive male rival when given a dichotomous choice in the current study. This lack of social group affiliation preference did not appear to be context dependent (i.e. female presence or absence), and males were equally attentive to both the rival male and the female within the stimulus shoals. The lack of social group preference is also not likely owing to our use of gravid, unreceptive females (rather than receptive ones) as stimulus fish in the paired stimulus shoals because Trinidadian male guppies are sexually attracted to both types of female [30, 31] and vigorously pursue, court and attempt to mate with gravid, unreceptive females [26, 30–32, 53].

As a resultant expectation from our aforementioned laboratory findings but contrary to our a priori prediction, free-ranging male guppies were not assorted by either body length or coloration within shoals in the Upper Aripo River. It is possible, however, that our field scan-sampling, ‘gambit-of-the group’ method [3] failed to detect phenotypic assortment among wild male guppies when it might have occurred. Alternative field methods, such as the capturing-recapturing of individually marked males within intact groups or tracking marked focal males (of known body length and coloration) in real time with video and the use of social network analysis of the data, might better reveal preferential associations among males based on phenotypic traits (e.g. [15, 21, 22]). Nonetheless, the highly dynamic fission-fusion nature of guppy societies [21–23] and extensive male mate-searching movements [26, 31] may constrain the expression of any male-male partner preferences to a set of restricted conditions in the wild. Males leave and join shoals much more frequently than females [21, 23] and their tenure in any given shoal is relatively brief (i.e. < 1 min) ([21], S.J. Potter, H.L. Auld, T.N. Sherratt and J.-G.J. Godin, unpublished data), which might limit opportunities for stable social associations to form. Although female guppies can form stable partner associations within shoals in the wild [22, 66], males either do not [22] or only weakly do so [66] depending on the population. This proposition is consistent with the results of our simulation model, which suggest that stable male-male associations based on body colour and(or) length phenotype is not likely to develop in a fission-fusion society when inter-group mobility among males is inversely related to their relative sexual attractiveness and(or) competitiveness and greater than that of females. If so, then the apparent lack of an evolved and expressed preference in wild male guppies from our study population to form social associations with other males based on their relative sexual attractiveness and competitiveness could be owing to the high fission-fusion dynamics of guppy shoals in nature. Such social dynamics likely places constraints on the formation of stable phenotype-based social associations among males. In comparison, in species populations such as the house finch [15] and fungus beetle [16] with seemingly lesser group fission-fusion dynamics than the guppy, individual males are able to select and remain in social groups in which their individual reproductive success is maximized. It is nonetheless possible that wild male guppies can maximize their mating success by non-randomly joining certain mixed-sex shoals over others using appropriate phenotype-dependent movement rules, but that such social preferences do not result in population structuring in nature.

The results of our current study with wild-caught guppies from a low-predation population in Trinidad differ from the recent laboratory findings of Gasparini et al. [18] who showed that laboratory-born and reared male guppies, originating from a different (high-predation) population than ours, preferentially associated with females that were surrounded by drabber rivals and that the magnitude of this preference was inversely correlated with the degree of the focal male’s colour ornamentation. The non-concordance between our current findings and theirs might be owing to methodological differences between the studies. First, the natural source populations of guppies for our study and that of Gasparini et al.’s [18] are different and have evolved under different predation regimes; predation risk is known to have shaped the evolution of shoaling or social affiliation behaviour [25, 29] and mating strategies and tactics [70, 71] in the guppy. Second, the study fish of Gasparini et al. [18] were laboratory-born and reared, whereas ours for the most part were wild-caught (Experiment 1) or free-ranging, and presumably had quite different prior social experiences. Lastly, Gasparini et al. [18] assessed male sexual attractiveness based on body coloration only, whereas we concurrently used body coloration and body length, both sexually-selected traits in the guppy. A male’s assessment of his own sexual attractiveness and that of his sexual rivals is presumably acquired through prior social experiences with rival males and prior sexual interactions with females [18, 36, 72], which likely differed between their lab-reared guppies and our wild-caught and free-ranging fish, as mentioned above.

In conclusion, our results collectively showed that male guppies from a natural low-predation population in Trinidad did not express any preference to affiliate with mixed-sex social groups on the basis of their relative sexual attractiveness and(or) sexual competitiveness in the laboratory and that males within free-ranging shoals in nature were not assorted by body colour or length, perhaps because of their high inter-group mobility and the highly dynamic fission-fusion nature of their societies. If wild male guppies in our study population had been negatively assorted by body colour and(or) length (i.e. more phenotypically different within than between shoals), as would be expected if they had been actively avoiding to associate with male rivals that were more sexually attractive and(or) sexual competitive than themselves [14, 18, 19], then such a non-random pattern of association could potentially weaken directional sexual selection generated by female preference for more colourful and larger males and thereby serve as a mechanism contributing to the maintenance of existing population-level variation in colour and body length in males (cf. Fig 2). Notwithstanding the recent complementary study of Gasparini et al. [18] on the guppy, the strength of our current study is that it comprehensively incorporated laboratory, field and modeling components to test a specific hypothesis, derived from sexual and social selection theories, about socio-sexual environment preferences and their implications for male phenotypic assortment within social groups. Although negative, our novel findings nonetheless importantly contribute to a growing body of work on the influence of the social environment in shaping individual behaviour, with implications for sexual selection and social evolution [4–8, 15–18], and should encourage further research on phenotype-dependent male movement and social association strategies across a range of societies with different fission-fusion dynamics [24, 25].

## Supporting Information

S1 Fig Phenotypic traits of control male guppies. Mean (± SE) body lengths and body colour scores of the focal males and the less attractive and more attractive stimulus males in the paired stimulus shoals for the control Experiment 2. For each panel, the differences among means were compared using the ANOVA, following by the Tukey HSD test for multiple comparisons of means. All pairwise means of both body length and colour score were significantly different from each other (*p* < 0.001), as indicated by different letters above the histogram bars.
(TIF)

S2 Fig Field study site on the Upper Aripo River. Schematic diagram (not to scale) of pools sampled for mixed-sex shoals of guppies in the Upper Aripo River, Trinidad. The identification number (P1–P6), maximum linear dimensions (width x length, in meters), and the distances (m) between each of the six sampled pools are indicated. Shallow stream riffle sections separated adjacent pools. Arrows indicate the direction of water flow.
(TIF)

S3 Fig Sensitivity analysis of the simulation model with variation in the number of males in the population. Results of a sensitivity analysis of the simulation model, testing for the model’s sensitivity to systematic variation in the number of males (*N*) in the ‘population’ whilst keeping all other parameters constant (i.e. ∝ = 0, *ß*_*C*_ = 0, *ß*_*S*_ = 0, *ß*_*R*_ = 1). Shown above are the relationships between the mean (± SE) regression coefficient for within-group variances in male body coloration (a) and body length (b) and varying number of males.
(TIF)

S4 Fig Sensitivity analysis of the simulation model with variation in the movement parameters for a population of 50 males. Results of a sensitivity analysis of the simulation model, testing for the model’s sensitivity to variation in the phenotype-based movement parameters *ß*_*C*_ (betac) and *ß*_*S*_ (betas) independently for *N* = 50 males in the ‘population. When *ß*_*C*_ is varied, *ß*_*S*_ is kept constant at 0, and vice versa; all other parameters are kept constant. Shown above are the relationships between the mean (± SE) regression coefficient for within-group variances in male body coloration (a, c) and body length (b, d) and varying parameters *ß*_*C*_ and *ß*_*S*_, respectively.
(TIF)

S5 Fig Sensitivity analysis of the simulation model with variation in the movement parameters for a population of 170 males. Results of a sensitivity analysis of the simulation model, testing for the model’s sensitivity to variation in the phenotype-based movement parameters *ß*_*C*_ (betac) and *ß*_*S*_ (betas) independently for *N* = 170 males in the ‘population’. The remainder of the caption is similar to that of the caption for S4 Fig.
(TIF)

S6 Fig Sensitivity analysis of the simulation model with variation in the movement parameters for a population of 500 males. Results of a sensitivity analysis of the simulation model, testing for the model’s sensitivity to variation in the phenotype-based movement parameters *ß*_*C*_ (betac) and *ß*_*S*_ (betas) independently for *N* = 500 males in the ‘population’. The remainder of the caption is similar to that of the caption for S4 Fig.
(TIF)
