# Supplementary figures and images for: Do Males Form Social Associations Based on Sexual Attractiveness in a Fission-Fusion Fish Society?
Source: PLoS One. 2016 Mar 17;11(3):e0151243. doi: 10.1371/journal.pone.0151243 (PMC4795762; doi:10.1371/journal.pone.0151243)

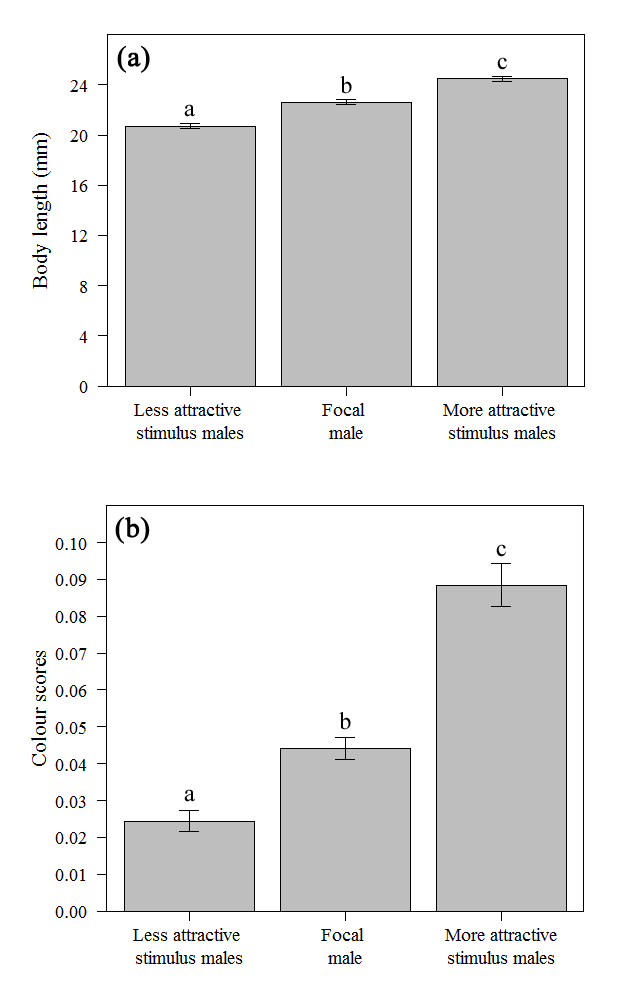

Supplement: S1 Fig — Mean (± SE) body lengths and body colour scores of the focal males and the less attractive and more attractive stimulus males in the paired stimulus shoals for the control Experiment 2. For each panel, the differences among means were compared using the ANOVA, following by the Tukey HSD test for multiple comparisons of means. All pairwise means of both body length and colour score were significantly different from each other (p < 0.001), as indicated by different letters above the histogram bars. (TIF) [file pone.0151243.s001.tif]

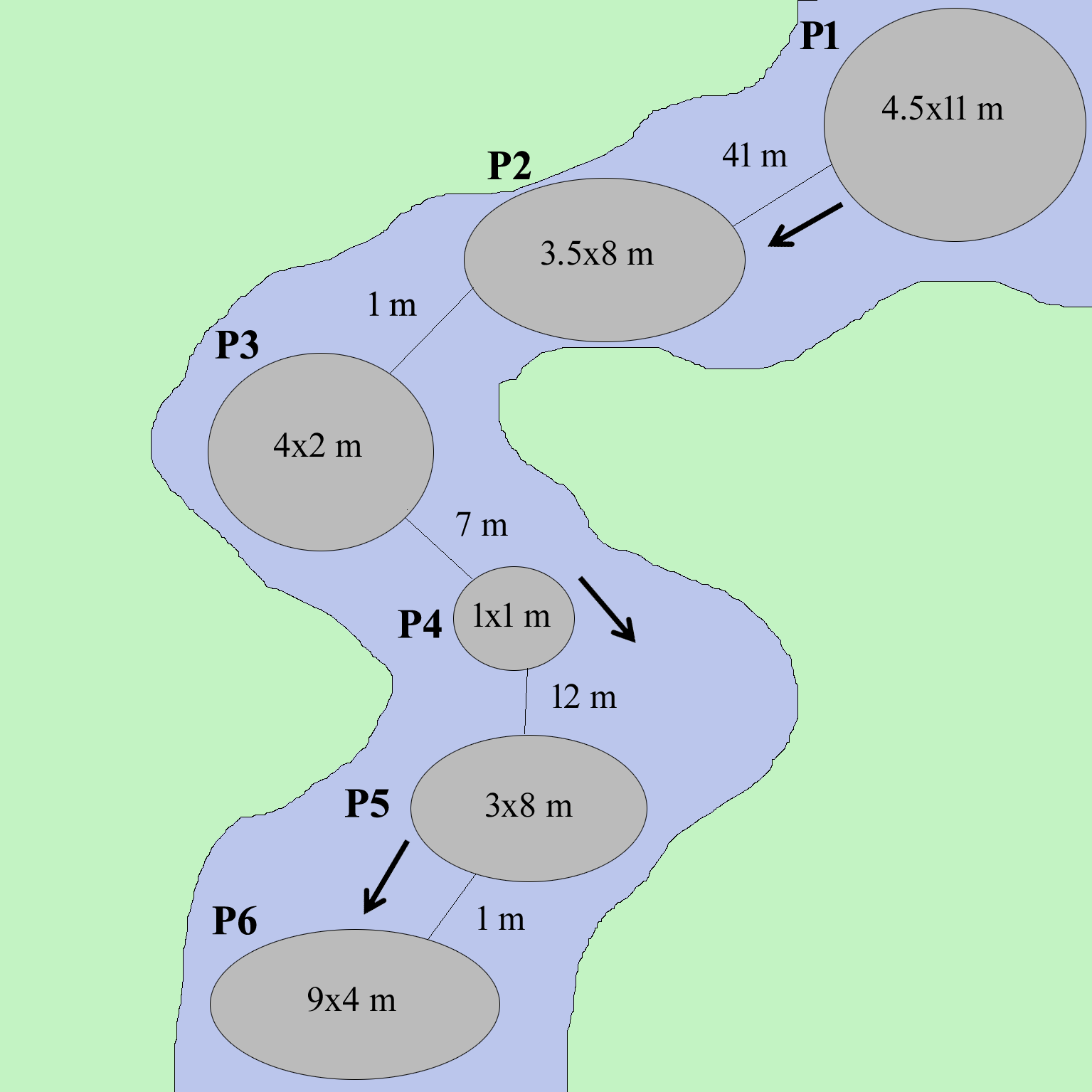

Supplement: S2 Fig — Schematic diagram (not to scale) of pools sampled for mixed-sex shoals of guppies in the Upper Aripo River, Trinidad. The identification number (P1–P6), maximum linear dimensions (width x length, in meters), and the distances (m) between each of the six sampled pools are indicated. Shallow stream riffle sections separated adjacent pools. Arrows indicate the direction of water flow. (TIF) [file pone.0151243.s002.tif]

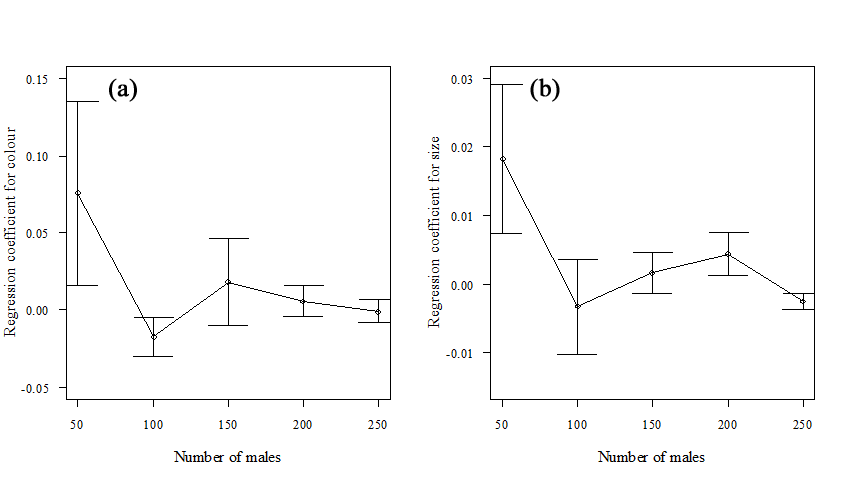

Supplement: S3 Fig — Results of a sensitivity analysis of the simulation model, testing for the model’s sensitivity to systematic variation in the number of males (N) in the ‘population’ whilst keeping all other parameters constant (i.e. ∝ = 0, ßC = 0, ßS = 0, ßR = 1). Shown above are the relationships between the mean (± SE) regression coefficient for within-group variances in male body coloration (a) and body length (b) and varying number of males. (TIF) [file pone.0151243.s003.tif]

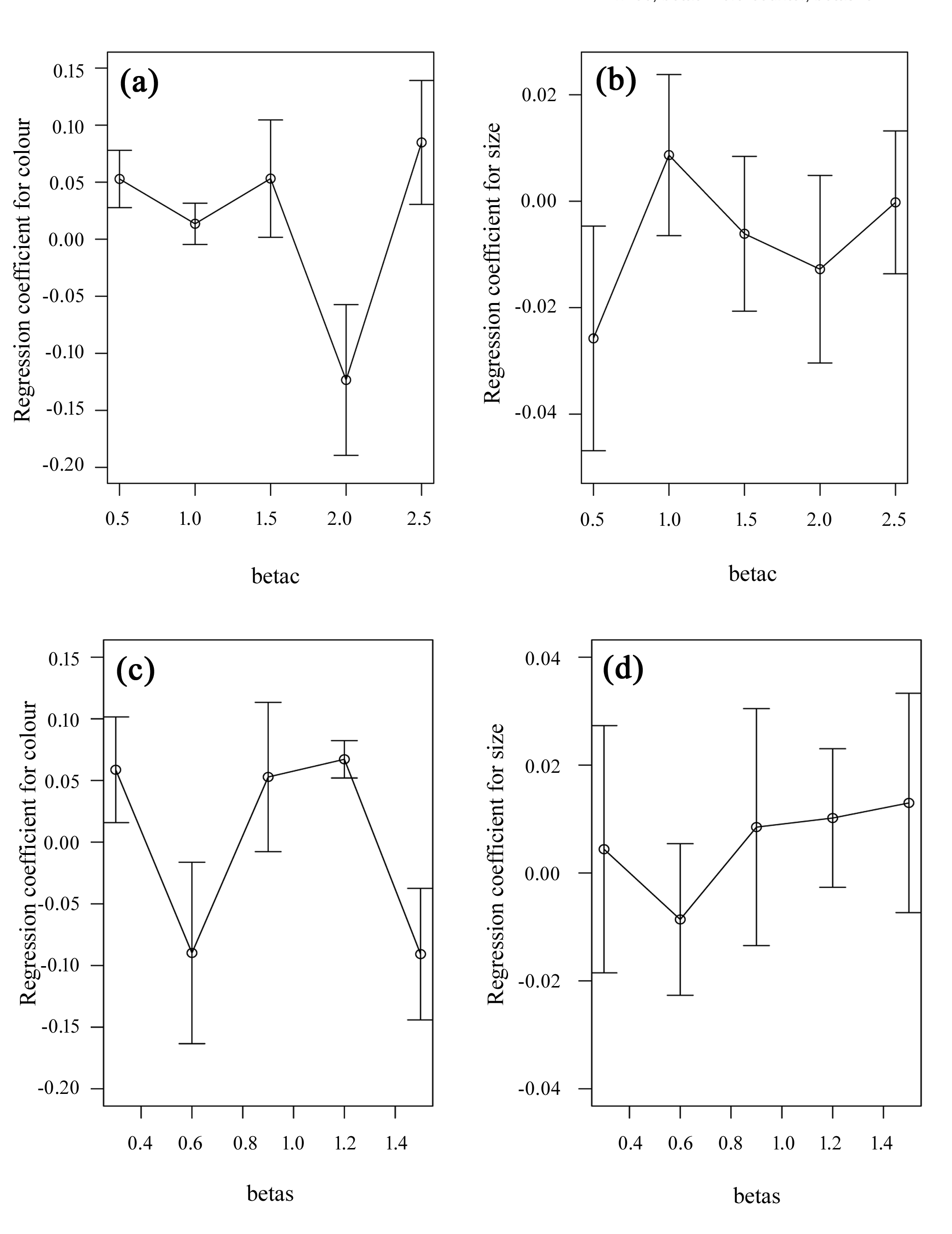

Supplement: S4 Fig — Results of a sensitivity analysis of the simulation model, testing for the model’s sensitivity to variation in the phenotype-based movement parameters ßC (betac) and ßS (betas) independently for N = 50 males in the ‘population. When ßC is varied, ßS is kept constant at 0, and vice versa; all other parameters are kept constant. Shown above are the relationships between the mean (± SE) regression coefficient for within-group variances in male body coloration (a, c) and body length (b, d) and varying parameters ßC and ßS, respectively. (TIF) [file pone.0151243.s004.tif]

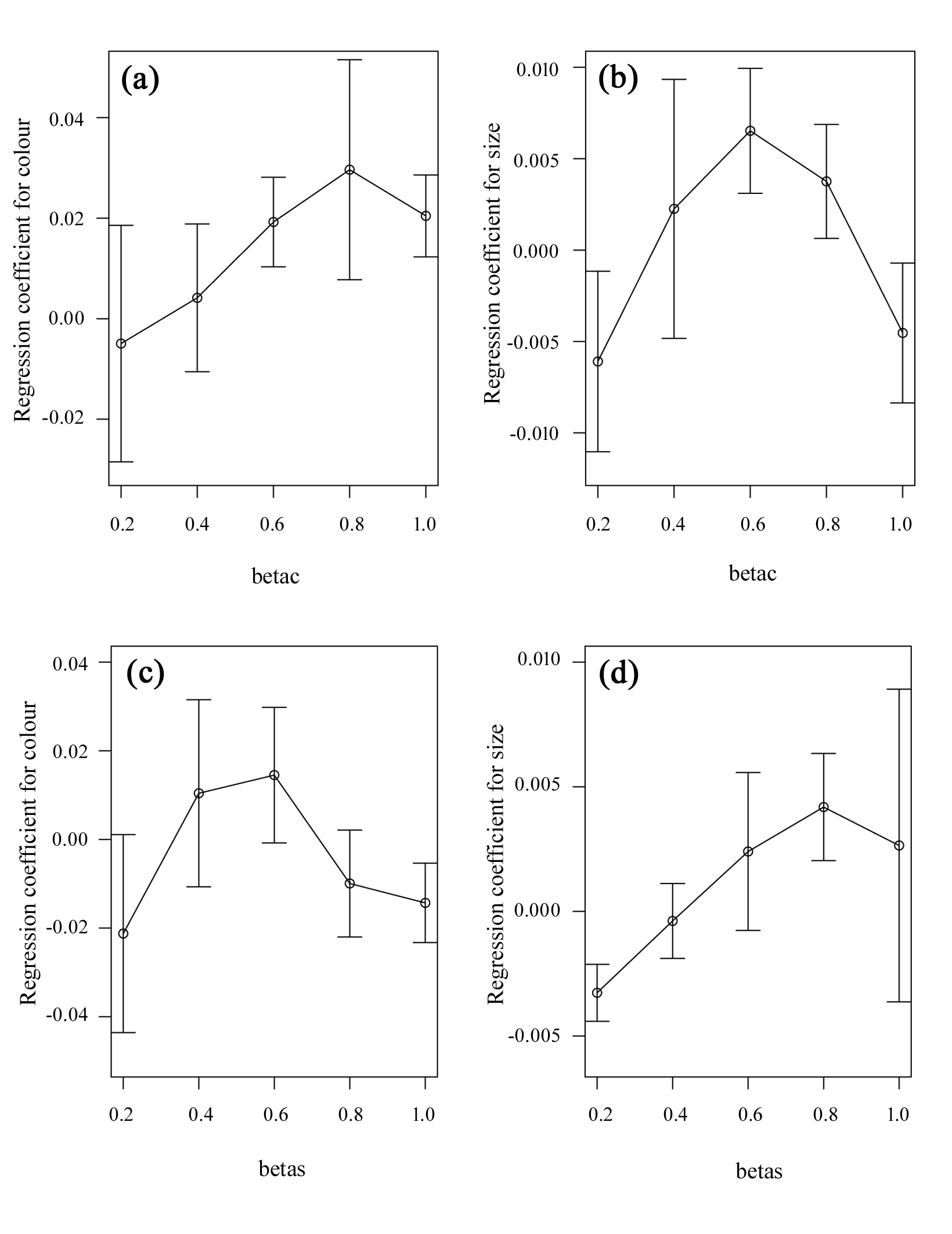

Supplement: S5 Fig — Results of a sensitivity analysis of the simulation model, testing for the model’s sensitivity to variation in the phenotype-based movement parameters ßC (betac) and ßS (betas) independently for N = 170 males in the ‘population’. The remainder of the caption is similar to that of the caption for S4 Fig. (TIF) [file pone.0151243.s005.tif]

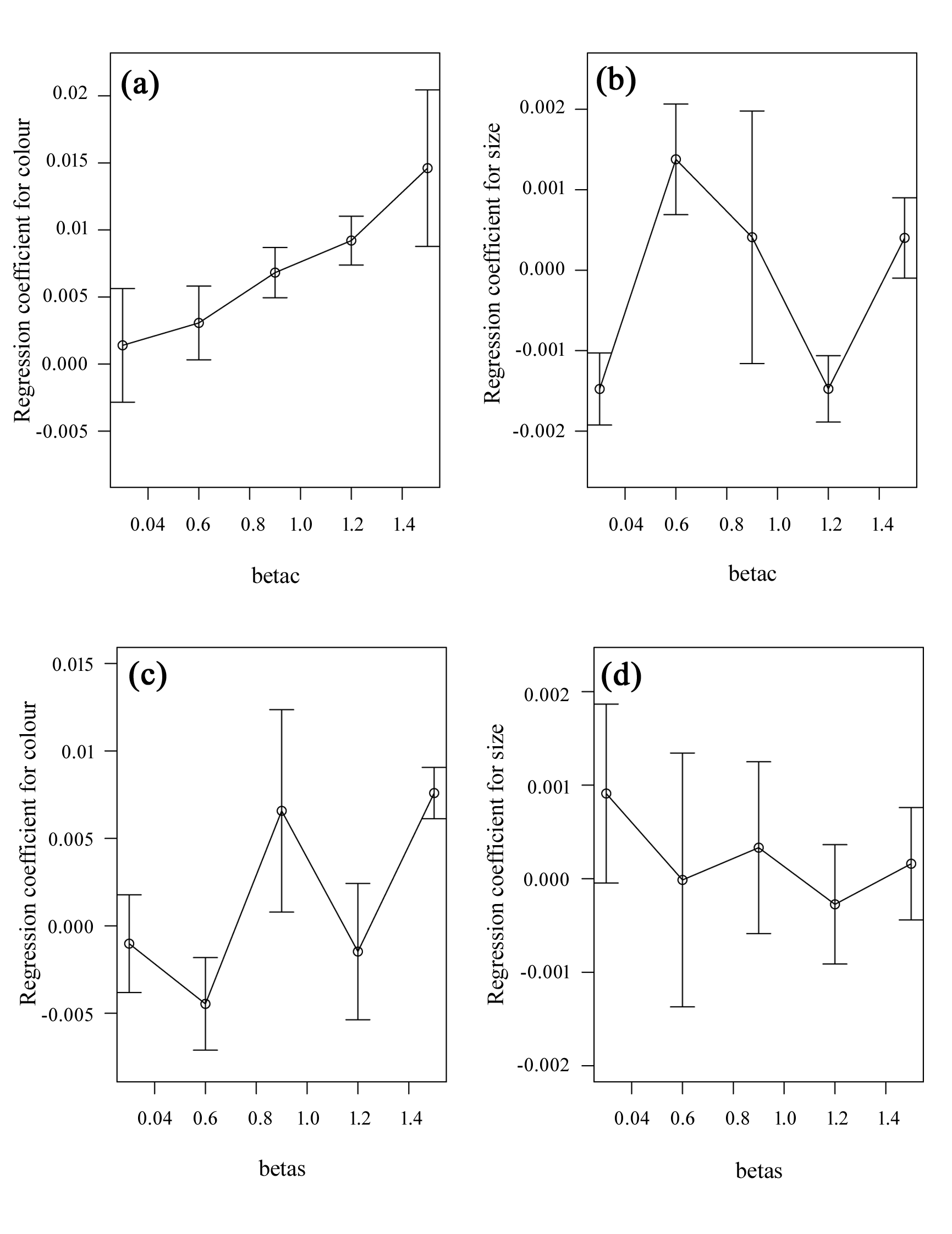

Supplement: S6 Fig — Results of a sensitivity analysis of the simulation model, testing for the model’s sensitivity to variation in the phenotype-based movement parameters ßC (betac) and ßS (betas) independently for N = 500 males in the ‘population’. The remainder of the caption is similar to that of the caption for S4 Fig. (TIF) [file pone.0151243.s006.tif]
